# Depression, anxiety, and smartphone addiction in university students- A cross sectional study

**DOI:** 10.1371/journal.pone.0182239

**Published:** 2017-08-04

**Authors:** Jocelyne Matar Boumosleh, Doris Jaalouk

**Affiliations:** Department of Nursing and Health Sciences, Notre Dame University-Louaize, Zouk Mosbeh, Lebanon; Tokyo Institute of Technology, JAPAN

## Abstract

**Objectives:**

The study aims to assess prevalence of smartphone addiction symptoms, and to ascertain whether depression or anxiety, independently, contributes to smartphone addiction level among a sample of Lebanese university students, while adjusting simultaneously for important sociodemographic, academic, lifestyle, personality trait, and smartphone-related variables.

**Methods:**

A random sample of 688 undergraduate university students (mean age = 20.64 ±1.88 years; 53% men) completed a survey composed of a) questions about socio-demographics, academics, lifestyle behaviors, personality type, and smartphone use-related variables; b) 26-item Smartphone Addiction Inventory (SPAI) Scale; and c) brief screeners of depression and anxiety (PHQ-2 and GAD-2), which constitute the two core DSM-IV items for major depressive disorder and generalized anxiety disorder, respectively.

**Results:**

Prevalence rates of smartphone-related compulsive behavior, functional impairment, tolerance and withdrawal symptoms were substantial. 35.9% felt tired during daytime due to late-night smartphone use, 38.1% acknowledged decreased sleep quality, and 35.8% slept less than four hours due to smartphone use more than once. Whereas gender, residence, work hours per week, faculty, academic performance (GPA), lifestyle habits (smoking and alcohol drinking), and religious practice did not associate with smartphone addiction score; personality type A, class (year 2 vs. year 3), younger age at first smartphone use, excessive use during a weekday, using it for entertainment and not using it to call family members, and having depression or anxiety, showed statistically significant associations with smartphone addiction. Depression and anxiety scores emerged as independent positive predictors of smartphone addiction, after adjustment for confounders.

**Conclusion:**

Several independent positive predictors of smartphone addiction emerged including depression and anxiety. It could be that young adults with personality type A experiencing high stress level and low mood may lack positive stress coping mechanisms and mood management techniques and are thus highly susceptible to smartphone addiction.

## Introduction

A smartphone is “a mobile phone that performs many of the functions of a computer, typically having a touch screen interface, internet access, and an operating system capable of running downloaded applications” [1]. These features have made smartphone use/ ownership a prevalent social phenomenon. According to a recent survey conducted by an opinion polling organization (IPSOS-STAT), smartphone penetration across total population in Lebanon has increased tremendously from 36% in 2012 to 70% in 2014, with Lebanon having the second highest growth rate (+34% points) among five Arab countries with available data (Kuwait: +37% points, UAE: +30% points, Saudi Arabia: +16% points, Egypt: +7% points). In addition, the proportion of smartphone owners with internet access on their device in Lebanon has increased by 16 percentage points between 2012 and 2014 (74% in 2012 to 90% in 2014), the second highest increase among the five Arab countries (UAE: +19% points, Saudi Arabia: +16% points, Kuwait: +12% points, Egypt: - 4% points) [2].

These technological changes have led to a revision in the very definition of addiction for it not only refers to drug or substance abuse, but now also includes behavioral addictions such as gambling, internet gaming, or even excessive smartphone use. For instance, the revised chapter of “Substance-Related and Addictive Disorders” in the fifth edition of the Diagnostic and Statistical Manual of Mental Disorders (DSM-5) includes a behavior-related condition “pathological gambling” as a diagnosable addictive disorder rather than an “impulse control disorder” in a new category on “behavioral addictions” [3]. In addition, “Internet Gaming Disorder” is listed in DSM-5 section III as a problematic behavior pending more research before considering it as a formal addictive disorder [4]. Although evidence-based research was not sufficient for smartphone addiction to be included in the DSM-5, a growing number of studies are confirming that habitual smartphone use is associated with several addictive characteristics that are analogous to symptoms of substance-use disorder as per DSM-5 including preoccupation, tolerance, inability to control craving, impairment of daily life functions, disregard to harmful consequences, and withdrawal.

A cross-sectional study conducted among a sample of 197 company employees and university students (age range = 18–53 years; mean age = 26.1 years) from South Korea, using Smartphone Addiction Scale (SAS), revealed six smartphone addictive symptoms−daily-life disturbance, positive anticipation, withdrawal, cyberspace-oriented relationship, overuse, and tolerance [5]. Another survey of a nationally representative South Korean sample of 795 school students (elementary to high school), using Smartphone Addiction Proneness Scale (SAPS) for youth, identified four smartphone addictive symptoms − disturbance of adaptive functions, virtual life orientation, withdrawal, and tolerance [6]. Similar findings were reported in studies done in Taiwan and China. Four smartphone-related addictive symptoms − compulsive behavior, functional impairment, tolerance and withdrawal− emerged from a survey of 283 university students from Taiwan (mean age = 22.9 years), using a newly developed Smartphone Addiction Inventory Scale (SPAI) [7]. Likewise, using a composite Smartphone Addiction Index, five smartphone addiction symptoms − disregard of harmful consequences, preoccupation, inability to control craving, productivity loss, and feeling anxious and lost− emerged among a sample of 414 Chinese university students aged 19–26 years [8].

Several other studies have also investigated the prevalence of smartphone addictive behaviors among young age groups. Given that they employed different methods to assess smartphone addiction, it might be difficult to compare prevalence figures reported in these studies. In Asia, a study involving 210 Korean female university students (mean age = 22 years) revealed that 30.5% had high risk to smartphone addiction [9]. Another study done in Korea by the Korean Ministry of Gender Equality and Family in 2013 reported that 17.9% of Korean adolescents showed smartphone addiction [10]. A study conducted among 414 Chinese university students (aged 19–26 years) identified 13.5% of the sample as smartphone addicts [8]. Another study conducted in Turkey revealed that 39.8% of a sample of 319 Turkish university students (mean age = 20.5 years) were excessive smartphone users (had Smartphone Addiction Scale (SAS) scores ≥ median score) [11]. In a single study done in Lebanon, 44.6% of 249 private university Lebanese students (mean age = 20.96 years) were found to be at high risk of smartphone addiction [12]. In the US, among a sample of 200 Stanford university students 10% and 34% acknowledged full addiction and almost addiction to iPhones, respectively [13]. Likewise, 11.2% of 276 African American college students (age range = 17–30) showed high level of smartphone addiction (≥ 90^th^ percentile SAS-SV score) [14].

While few studies examined the independent predictive effect of depression and anxiety on smartphone addiction in college students, they fell short of controlling, at the same time, for multiple sociodemographic, academic, lifestyle, personality traits, religious practice, and smartphone-related variables (age at first use, duration of use per weekday, purpose of using smartphone) in the studied sample [11,15–17]. In other words, when investigators adjusted for the effects of confounding variables when assessing the independent contribution of depression or anxiety to smartphone addiction in university students, it was limited to isolated sociodemographic and/ or academic, or smartphone use-related variables. Given the high penetration rate of smartphone in Lebanon; the association of smartphone use with addiction and undesirable health effects; and the likelihood that smartphone addiction may have depression or anxiety as underlying independent risk factors; hence, it becomes important to quantify smartphone addiction/ smartphone-related addictive symptoms and assess possible contribution of depression or anxiety to smartphone addiction in Lebanese students. This study aims to 1) assess prevalence of smartphone addiction symptoms, in relation to physical, mental and social health, and 2) investigate whether depression or anxiety, independently, contributes to smartphone addiction level among a sample of Lebanese students, while simultaneously adjusting for other independent variables.

## Methods

A total of 688 undergraduate students at Notre Dame University (NDU), Lebanon were recruited from the pool of “Natural Sciences”, general education requirement courses (GERs). GERs include a relatively random sample of students from each of NDU’s seven faculties and students are required to take one 3- credit course from this pool. Students were selected by simple random sampling method and invited via email to participate in the study. In the email, researchers briefed the students about the study’s objectives and procedures. Those who agreed to participate (92.5%) were then invited, via an online system, to sign a consent form and complete a self-administered survey constructed of two sections. One section included questions on socio-demographics (age, gender, income, residence, hours of work per week), academics (cumulative grade-point-average (GPA), class, major, faculty), smartphone use (age at first use of smartphone, duration/ hours of smartphone use during a weekday, reason/ purpose for using smartphone), personality type, and select lifestyle behaviors (smoking, alcohol drinking, religious practice). With regard to personality type, participants were asked to respond to the following question included in the self administered survey: Identify your personality type: 1) type A (aggressive, competitive, angry, cynical, mistrustful), or 2) type B (easy going, laid-back/ more relaxed), patient). The other section included a 26-item Smartphone Addiction Inventory (SPAI) Scale [7]. The SPAI scale is comprised of four subscales: compulsive behavior (CB; 9 items), functional impairment (FI; 8 items), withdrawal (W; 6 items) and tolerance (T; 3 items). The Cronbach's alpha for the total SPAI scale was 0.94 [7]. Participants were asked to rate items on a 4-point Likert scale, 1 = “strongly disagree”, 2 = “somewhat disagree”; 3 = “somewhat agree”; 4 = “strongly agree” so that the total SPAI score (Addiction Score) ranges from 26 to 104. Excessive smartphone use was defined by at least 5 hours (median number of hours spent using smartphone) on a weekday. Of the 688 students, 634, 463 and 417 students were also asked questions on lifestyle habits (smoking, alcohol drinking), personality type, reason for smartphone use and current place of stay; frequency of religious practice; and depression and anxiety, respectively. Brief screeners of depression and anxiety (PHQ-2 and GAD-2), two screeners that are often used in research settings, were used [18–20]. These consist of the first two items of the Patient Health Questionnaire-9 (PHQ-9), a 9-item depression scale, and the Generalized Anxiety Disorder-7 (GAD-7), a 7-item anxiety scale, respectively, and constitute the two core DSM-IV items for major depressive disorder and generalized anxiety disorder, respectively. Each of the items is scored 0 to 3, providing a 0 to 6 total score. A score of 3 or greater, the recommended cut-point for each when used as a screener, was used to screen for depression and anxiety in our study [21]. Information on socio-demographic, academic and lifestyle variables was collected from non-responders and descriptive statistics were performed to ensure that responders and non-responders were similar on all these factors. Based on an estimated prevalence of smartphone addiction of about 15% among university students in Lebanon, the sample size was calculated and found to be approximately 196. Collection of data took place during Fall 2014 and Spring 2015 academic terms. Data were collected in an anonymous manner (i.e. no names, identification numbers, or any other personal identifiers were requested) to avoid participants’ attempt to hide sensitive information from researchers. All data forms were maintained in locked cabinets in the researchers’ offices and access was strictly limited to study investigators. Likewise, computerized data were stored on a password protected computer in a locked office of the research team. The study was approved by the research ethics committee at NDU called “Institutional Review Board at NDU”.

Descriptive statistics for the total sample were performed. Quantitative and qualitative measurements were summarized as mean ± standard deviation and n (%), respectively. We performed comparisons of continuous and categorical variables by using independent 2-sample T Test/ Mann-Whitney-U-test/ analysis of variance and the chi square test /Fisher's exact test, respectively. Multiple linear regression was used to assess the relationship between mental health problems (depression, anxiety) and smartphone addiction level (total SPAI score), after controlling for the effects of confounders. Preliminary analyses were conducted to ensure no violation of the assumptions of normality, linearity, multicollinearity and homoscedasticity. Covariates that were found to be associated with addiction level (total SPAI score) at p *<* 0.05 level in the bivariate analyses were entered into the models. Model 1 was unadjusted, showing the main effect of depression/anxiety (independent variable) on smartphone addiction level (total SPAI score-dependent variable); Model 2 was adjusted for age, personality type, and class. Model 3 was our fully adjusted model in which age, personality type, class, age at first use of smartphone, duration of smartphone use during a weekday and reasons for smartphone use were controlled for. In our analyses, missing values were excluded case by case/ pairwise. Spearman correlation coefficients were used to evaluate the association among the different variables. Statistical analyses were performed using the Statistical Package for Social Sciences (SPSS) version 22 for Windows. A p-value less than 0.05 was considered statistically significant.

## Results

The sample consisted of 688 undergraduate students (53% men and 47% women) with a mean age of 20.64±1.88 years. Their mean age at first use of smartphone was found to be 15.09±2.12 years, with about 49% reporting excessive smartphone use (≥5 hours/weekday). About one-third of the students in our sample were smokers, three-fifths were alcohol drinkers, one-fifth were depressed, and one-fourth were anxious. The top three reasons for smartphone use were reported to be texting (83%), entertainment/calling family members (67%), and calling friends (62%) (Table 1).

**Table 1:** Characteristics of study participants (n = 688).

	[Mean ± SD (n)] Or n (%)		[Mean ± SD (n)] Or n (%)
**Age**	20.64 ± 1.88 (665)	**Smoking**	
**Faculty**		Yes	210 (33.7)
Architecture & Design	112 (16.7)	No	414 (66.3)
Business	213 (31.8)	**Alcohol drinking**	
Engineering	141 (21.0)	Yes	393 (62.5)
Humanities	126 (18.8)	No	236 (37.5)
Law & Political Sc.	11 (1.6)	**Religious practice**	
Natural & Applied Sc.	56 (8.4)	Daily	120 (26.0)
Health Sc.	11 (1.6)	Once or several times a week	146 (31.7)
**Class**		Once or several times a month	84 (18.2)
Sophomore (Year 1)	268 (39.4)	A few times a year	81 (17.6)
Junior (Year 2)	231 (33.9)	Not at all	30 (6.5)
Senior (Year 3)	182 (26.7)	**Age at first use of smartphone**	15.09 ± 2.12 (665)
**GPA (Grade Point Average)**		**Total SPAI Score**	55.37 ± 15.08 (460)
≤ 1.99	46 (6.7)	**Duration of smartphone use/weekday**	
2.00–2.99	461 (68.4)	0 to less than 1 hour	14 (2.1)
≥ 3.00	168 (24.9)	1 to less than 2 hours	70 (10.3)
**Work hours per week**		2 to less than 3 hours	55 (8.1)
0 hours	196 (29)	3 to less than 4 hours	133 (19.5)
1–10 hours	220 (32)	4 to less than 5 hours	78 (11.5)
11–15 hours	94 (14)	≥ to 5 hours	331 (48.6)
> 15 hours	171 (25)	**Reason for using smartphone**	
**Residence at present time**		Calling family members (n = 634)	421 (67)
Staying with family	520 (83)	Calling friends (n = 634)	387 (62)
Staying at one's apartment	26 (4)	Texting (n = 634)	519 (83)
Staying in dormitory	52 (8)	Entertainment (n = 634)	431 (67)
Other	32 (5)	Reading News (n = 634)	290 (46)
**Personality type**		Other (n = 634)	164 (26)
Type A	215 (34.4)	Study-related purposes (n = 417)	224 (54)
Type B	410 (65.6)	**Depressed**	89 (21.8)
		**Anxious**	108 (26.5)

More than one-fourth of the students in our sample reported indications of compulsive behavior (e.g., 38.5% reported that surfing the smartphone has exercised negative effects on their physical health). More than one-fifth of the sample reported indications of functional impairment (e.g., 40.7%, and 35.8% reported that their interaction with family members is decreased on account of smartphone use, and using smartphone has exercised certain negative effects on their schoolwork or job performance, respectively). More than one-sixth of the sample reported indications of withdrawal (e.g., 63.5% reported that the idea of using smartphone comes as the first thought on mind when waking up each morning). More than two-fifths of the sample reported indications of tolerance (e.g. 54.3% reported that they were told more than once that they spent too much time on smartphone) (Table 2).

**Table 2:** Prevalence of smartphone addiction (SPAI) symptoms among study participants (n = 688).

	Symptom	n (%)
**Compulsive Behavior (9 items)**	Although using Smartphone has brought negative effects on my interpersonal relationships, the amount of time spent on Internet remains unreduced	249 (37.5)
	I feel distressed or down once I cease using Smartphone for a certain period of time	195 (29.6)
	My life would be joyless hadn’t there been Smartphone	187 (29.9)
	My recreational activities are reduced due to Smartphone use	173 (27.5)
	I use Smartphone for a longer period of time and spend more money than I had intended	233 (35.4)
	I try to spend less time on smartphone, but the efforts were in vain	211 (33.8)
	I feel very vigorous upon Smartphone use regardless of the fatigues experienced	260 (39.3)
	Surfing the Smartphone has exercised negative effects on my physical health. For example, viewing Smartphone when crossing the street; fumbling with one’s Smartphone while driving or waiting, and resulted in danger	238 (38.5)
	I fail to control the impulse to use Smartphone	230 (35.2)
**Functional Impairment (8 items)**	I feel aches and soreness in the back or eye discomforts due to excessive Smartphone use	283 (43.5)
	I feel tired on daytime due to late-night use of Smartphone	223 (35.9)
	I make it a habit to use Smartphone and the sleep quality and total sleep time decreased	234 (38.1)
	I have slept less than four hours due to using Smartphone more than once	240 (35.8)
	To use Smartphone has exercised certain negative effects on my schoolwork or job performance	230 (35.8)
	I find myself indulged on the Smartphone at the cost of hanging out with friends	136 (21.2)
	My interaction with family members is decreased on account of Smartphone use	258 (40.7)
	I need to spend an increasing amount of time on Smartphone to achieve same satisfaction as before	173 (27.7)
**Withdrawal (6 items)**	I feel restless and irritable when the Smartphone is unavailable	268 (39.9)
	I feel uneasy once I stop Smartphone for a certain period of time	217 (32.4)
	I cannot have meal without Smartphone use	118 (18.9)
	The idea of using Smartphone comes as the first thought on mind when waking up each morning	409 (63.5)
	I feel missing something after stopping Smartphone for a certain period of time	294 (46.2)
	I feel the urge to use my Smartphone again right after I stopped using it	267 (42.8)
**Tolerance (3 items)**	I find that I have been hooking on Smartphone longer and longer	330 (49.5)
	I have increased substantial amount of time using Smartphone per week in recent 3 months	269 (41)
	I was told more than once that I spent too much time on Smartphone	366 (54.3)

Higher total SPAI score was found to be significantly associated with belonging to the Junior class (mean SPAI score: Junior 58.91 vs. Senior 53.28, p = 0.004), type A personality (mean SPAI score: type A 59.38 vs. type B 53.74, p = 0.000), excessive smartphone use (mean SPAI score: ≥ 5 hrs /weekday 60.61 vs. < 5 hrs/weekday 50.63, p = 0.000), use of smartphone for entertainment (mean SPAI score: users 57.05 vs. non- users 52.67, p = 0.011) and other (mean SPAI score: users 58.80 vs. non- users 54.64, p = 0.015) purposes, and non-use of smartphone for calling family members (mean SPAI score: users 54.71 vs. non-users 57.97, p = 0.044) and correlated with younger age at first use of smartphone (ρ = -0.168, p = 0.000). Higher total SPAI score was also found to be significantly associated with depression (mean SPAI score: depressed 61.11 vs. non-depressed 54.73, p = 0.004) and anxiety (mean SPAI score: anxious 59.04 vs. non- anxious 54.62, p = 0.028 (Table 3).

**Table 3:** Associations between smartphone addiction level (total SPAI score) and participants' characteristics.

	Addiction Score: Mean ± SD (n)		P-value
**Gender**			0.158
Male	54.45 ± 15.65 (245)		
Female	56.45 ± 14.26 (209)		
**Faculty**			0.798
Architecture & Design	57.22 ± 14.96 (55)		
Business	55.85 ± 16.00 (81)		
Engineering	55.67 ± 15.27 (57)		
Humanities	55.02 ± 13.00 (47)		
Law & Political Sc.	57.67 ± 12.88 (6)		
Natural & Applied Sc.	54.46 ± 15.09 (24)		
Health Sc.	68.67 ± 8.74 (3)		
**Class**			0.044
Sophomore	55.26 ± 13.13(113)		
Junior	58.91 ± 16.37 (94)*		
Senior	53.28 ± 15.35 (71)*		
**GPA (Grade Point Average)**			0.081
< 1.99	49.00 ± 14.71(23)		
2–2.99	56.10 ± 15.04 (307)		
> 3	54.72 ± 15.17(123)		
**Work hours per week**			0.574
0 hours	55.12 ± 14.76 (132)		
1–10 hours	56.48 ± 15.40 (155)		
11–15 hours	55.60 ± 13.84 (60)		
> 15 hours	53.86 ± 15.76 (111)		
**Residence at present time**			0.670
Staying with family	55.56 ± 15.19 (332)		
Staying at one's apartment	53.30 ± 19.68 (20)		
Staying in dormitory	57.62 ± 13.11 (37)		
Other	58.05 ± 15.49 (19)		
**Personality type**			0.000
Type A	59.38 ± 15.36 (147)		
Type B	53.74 ± 14.63 (259)		
**Smoking**			0.196
Yes	57.05 ± 16.14 (136)		
No	54.99 ± 14.65 (269)		
**Alcohol drinking**			0.536
Yes	56.08 ± 15.20 (260)		
No	55.11 ± 15.26 (150)		
**Religious practice**			0.255
≤ few times a year	54.30 ± 14.88 (82)		
≥ 1 time a month	56.53 ± 15.23 (229)		
**Duration of smartphone use/weekday**			0.000
≥5 hours	60.61 ± 13.36 (369)		
< 5 hours	50.63 ± 15.05 (88)		
**Reason for using smartphone**	**Yes**	**No**	
Calling family members (n = 634)	54.71 ± 15.50 (282)	57.97 ± 14.37 (128)	0.044
Calling friends (n = 634)	56.02 ± 15.12 (259)	55.23 ± 15.41 (151)	0.611
Texting (n = 634)	56.08 ± 14.94 (341)	53.99 ± 16.49 (69)	0.298
Entertainment (n = 634)	57.05 ± 14.47 (286)	52.67 ± 16.47 (196)	0.011
Reading News (n = 634)	56.83 ± 14.98 (196)	54.72 ± 15.39 (214)	0.162
Other (n = 634)	58.80 ± 14.41 (107)	54.64 ± 15.36 (303)	0.015
Study-related purposes (n = 417)	57.17 ± 14.74 (156)	54.48 ± 15.19 (122)	0.138
**Depression**	61.11 ± 14.45 (57)	54.73 ± 14.91 (215)	0.004
**Anxiety**	59.04 ± 15.29 (78)	54.62 ± 14.77 (196)	0.028

* p value pertains to difference between the 2 indicated groups

Multiple linear regression was used to assess the ability of two mental health problems (depression, anxiety) to predict addiction level (SPAI score), after controlling for the influence of confounding variables. In the unadjusted model (Model 1), higher addiction levels (total SPAI scores) were found to be significantly associated with higher depression /anxiety scores, whereby the total SPAI score increases by about 3 /1.7 units for each unit increase in depression / anxiety scores, and with depression/anxiety scores explaining about 7% and 2% of the variance in total SPAI scores, respectively. Age, personality type, and class were entered in Model 2, increasing the variance in total SPAI scores explained by the independent variables (depression/anxiety, age, personality type, and class) to 10%, and 7%, respectively. After additional entry of the independent variables that pertain to smartphone use habits in the final model (Model 3) (age at first use of smartphone, duration of smartphone use, and use of smartphone for calling family members, entertainment and other purposes), the total variance explained by the model as a whole increased to 23% and 21%, p = 0.000, respectively. In the final model in which depression score was entered as the main independent variable in model 1, higher total SPAI score was found to be significantly associated with higher depression score, personality type A, excessive smartphone use (≥ 5 hours/ weekday), non-use of smartphone for calling family members and use of smartphone for entertainment purposes, with excessive smartphone use recording the highest beta value (β = 0.262, p = 0.000) followed by depression score (β = 0.201, p = 0.000), non-use of smartphone to call family members (β = -0.148, p = 0.015), personality type A (β = 0.130, p = 0.019), and use of smartphone for entertainment purposes (β = 0.126, p = 0.035) (Table 4). Similarly, in the final model in which anxiety score was entered as the main independent variable in model 1, higher total SPAI score was found to be significantly associated with higher anxiety score, personality type A, excessive smartphone use, non-use of smartphone for calling family members, and use of smartphone for entertainment purposes, with excessive smartphone use recording the highest beta value (β = 0.268, p = 0.000) followed by non-use of smartphone to call family members (β = -0.160, p = 0.009), personality type A (β = 0.132, p = 0.020), use of smartphone for entertainment purposes (β = 0.125, p = 0.040), and anxiety score (β = 0.122, p = 0.034) (Table 5).

**Table 4:** Association between total SPAI score and depression score, as assessed by multiple linear regression*.

	Unstandardized β	S.E.	Standardized β	p-value	95% CI for unstandardized β		R square
					Lower Boundary	Upper Boundary	
**Model 1**:							0.066
Depression Score	3.078	0.707	0.256	0.000	1.687	4.469	
**Model 2**							0.103
Depression Score	2.806	0.708	0.234	0.000	1.412	4.200	
Age	-1.210	0.557	-0.150	0.031	-2.307	-0.113	
Personality type	4.568	1.860	0.144	0.015	0.906	8.231	
Class	1.038	1.305	0.055	0.427	-1.532	3.608	
**Model 3**							0.229
Depression Score	2.412	0.667	0.201	0.000	1.099	3.725	
Age	-0.921	0.563	-0.115	0.103	-2.030	0.188	
Personality type	4.109	1.744	0.130	0.019	0.675	7.543	
Class	1.005	1.231	0.054	0.415	-1.419	3.428	
Age at first use of smartphone	-0.554	0.443	-0.078	0.213	-1.427	0.319	
Duration of smartphone use/weekday	7.901	1.688	0.262	0.000	4.578	11.224	
Use of smartphone to call family members	-4.731	1.926	-0.148	0.015	-8.524	-0.939	
Use of smartphone for entertainment purposes	4.105	1.935	0.126	0.035	0.296	7.915	
Use of smartphone for other reasons	3.121	1.949	0.091	0.111	-0.718	6.959	

* Dependent Variable: Total Addiction Score

**Table 5:** Association between total SPAI score and anxiety score, as assessed by multiple linear regression*.

	Unstandardized β	S.E.	Standardized β	p-value	95% CI		R square
					Lower Boundary	Upper Boundary	
**Model 1**:							0.023
Anxiety Score	1.654	0.648	0.153	0.011	0.379	2.930	
**Model 2**							0.066
Anxiety Score	1.401	0.657	0.130	0.034	0.108	2.694	
Age	-1.353	0.568	-0.168	0.018	-2.470	-0.235	
Personality type	4.781	1.917	0.151	0.013	1.006	8.556	
Class	1.488	1.321	0.079	0.261	-1.113	4.089	
**Model 3**							0.205
Anxiety Score	1.320	0.620	0.122	0.034	0.098	2.541	
Age	-1.008	0.570	-0.125	0.078	-2.131	0.114	
Personality type	4.187	1.792	0.132	0.020	0.658	7.716	
Class	1.426	1.240	0.076	0.251	-1.016	3.867	
Age at first use of smartphone	-0.669	0.450	-0.094	0.138	-1.556	0.217	
Duration of smartphone use/weekday	8.068	1.708	0.268	0.000	4.705	11.430	
Use of smartphone to call family members	-5.118	1.945	-0.160	0.009	-8.948	-1.289	
Use of smartphone for entertainment purposes	4.073	1.971	0.125	0.040	0.191	7.954	
Use of smartphone for other reasons	3.358	1.974	0.098	0.090	-0.529	7.245	

* Dependent Variable: Total Addiction Score

## Discussion

Prevalence rates of smartphone-related compulsive behavior, functional impairment, tolerance and withdrawal symptoms were substantial. 35.9% of our sample reported that they felt tired during daytime due to late-night smartphone use, 38.1% of them acknowledged that their sleep quality is decreased, and 35.8% admitted that they slept less than four hours due to smartphone use more than once. This is in agreement with findings from other studies done among university and high school students. In a sample of 319 Turkish university students, smartphone addiction scores showed significant positive correlation with sleep disturbance, daytime dysfunction, subjective and global sleep quality scores [11]. Likewise, findings from a survey conducted among a convenient sample of 82 mid to high level managers enrolled in MBA classes revealed that smartphone use for work at night had negative effect on daytime work engagement mediated by sleep disruption and morning depletion of self-control resources [22]. A third study carried out among a sample of 362 Swiss high school students found out that adolescents with smartphones had delayed bedtimes and reported significantly more sleep difficulties and reduced sleep duration on weekdays compared to those with conventional mobile phones[23].

Whereas gender, residence, work hours per week, major field of study, academic performance (GPA), lifestyle habits such as smoking and alcohol drinking, and religious practice did not associate with smartphone addiction score; individual’s personality type (type A vs. type B), class (year 2 vs. year 3), smartphone-related variables [age at first use, excessive use of smartphone during a weekday, reason/ purpose of using smartphone (not using smartphone to call family members, entertainment, and other vs. calling friends, texting, reading news, and study-related purposes)], and having depression or anxiety, showed statistically significant associations with smartphone addiction. Using multiple regression analyses, the most powerful independent predictor of smartphone addiction turned out to be excessive use of smartphone during a weekday (5 or more hours during a weekday), followed by depression score, non-use of smartphone to call family members, personality type, use of smartphone for entertainment purposes, and anxiety score.

### Association of personality type and smartphone addiction

Individuals with type A personality are more competitive, ambitious, impatient, anxious, aggressive, and more likely to be workaholic. Individuals with type B personality are their counterparts. Type A behavior individuals are more likely to experience high stress level and have higher risk to develop ill health including cardiovascular disease and cancer, compared to type B behavior individuals. Our finding of a positive independent association of personality type A and smartphone addiction is congruent, by and large, with the results of several other studies which examined the link between personality traits and smartphone addiction. A survey conducted among a sample of 448 Korean university students revealed a significant positive association between neurotic personality trait and smartphone addiction severity level [10]. In another study surveying 353 Korean college students, both aggression and impulsion scores emerged as significant independent positive predictors of smartphone addiction, with impulsion being a stronger one [15]. Nonetheless, neurotic personality trait did not predict smartphone addiction in an African American sample of 276 college students [14].

We suppose that the positive relationship between personality type A and smartphone addiction severity is both direct and indirect, influenced by perceived high stress level. In a study carried out among a sample of 387 university students from Taiwan, high stress level (emotional, family, interpersonal, academic, or all combined) showed significant positive correlation with smartphone addiction [24]. In another survey of a sample of 274 adults (67.9% students), stress level showed significant positive association with smart device addiction [25].

### Associations of depression and anxiety and smartphone addiction

In our sample, depression and anxiety scores emerged as independent positive predictors of smartphone addiction, with depression score being a more powerful predictor compared to anxiety score. Our findings resonate well with prior results from multiple studies which looked at the relationship between psychological traits (depression, anxiety, social phobia, loneliness) and smartphone addiction. In a sample of 353 Korean college students, depression emerged as a significant independent positive predictor of smartphone addiction [15]. Mood regulation (defined as avoiding/ reducing negative feelings-loneliness, anxiety, depression, stress) had significant positive effect on smartphone addiction among a convenient sample of 394 Chinese university students [17]. Depressive state emerged as an independent predictor of immersion in Internet communication score in a survey of 126 Japanese medical university students [16]. In a survey of 414 Chinese university students, loneliness, which is highly positively associated with depression, emerged as the strongest independent predictor of smartphone addiction score [8]. Likewise, loneliness score showed significant positive correlation with smartphone addiction score and emerged as an independent predictor of cyberspace- oriented relationship score, in a sample of 367 Turkish university students [26]. Mean depression and anxiety scores were significantly higher among high versus low smartphone users, and emerged as independent predictors of smartphone addiction severity as per findings from a survey of 319 Turkish university students [11]. Social interaction anxiety, and social phobia, emerged as independent positive predictors of smartphone addiction in surveys of 276 African American [14], and 367 Turkish university students’ samples [26], respectively.

The link between depression or anxiety and smartphone addiction may not just be established among young adults/ university students; rather it may be applicable to the general adult population. In a mixed sample of 274 adults aged 16–59 years, depression and anxiety had significant positive correlation with smart device addiction [25]. Findings from a study among a sample of 325 Taiwanese adults (age range = 17–97 years), comparable in terms of age and gender to nationally representative sample, revealed a statistically significant positive effect of social interaction anxiety on smartphone-related compulsive use [27].

### Study strengths and limitations

While published literature took into account few of the important confounders when examining the independent association between depression/ anxiety and smartphone addiction, our study examined this association while controlling simultaneously for the effects of all these confounding variables (sociodemographic, academic, lifestyle habits, personality type, and smartphone-related variables). In addition, the tools that we employed for assessing smartphone addiction and screening depression and anxiety have been used and were validated among samples of university students.

The study employed a cross-sectional design hence identified significant relationships between tested independent variables and the dependent variable (smartphone addiction) cannot be inferred as causal. In addition, data on many tested independent variables were self-reported and may possibly bear some inaccuracies for not wanting to reveal vulnerabilities (even if data collection forms did not bear student’s personal identifiers) or because of recall bias (time spent and reason for using smartphone during a weekday, age at first use of smartphone).

## Conclusion

In conclusion, prevalence of smartphone addiction symptoms was substantial among our sample of university students. Several independent risk factors for smartphone addiction emerged including excessive use of smartphone, personality type A, depression, anxiety, and a possible lack of family social support (indicated by not calling family members). We posit that many of the identified risk factors may share an underlying causal variable which is high stress level. It could be that young adults with personality type A experiencing high stress level and low mood may lack positive stress coping and mood management techniques and are highly susceptible to smartphone addiction.

## Supporting information

S1 Dataset Raw data for all participants. (XLS)
